# Preventive Effects of a Fermented Dairy Product against Alzheimer’s Disease and Identification of a Novel Oleamide with Enhanced Microglial Phagocytosis and Anti-Inflammatory Activity

**DOI:** 10.1371/journal.pone.0118512

**Published:** 2015-03-11

**Authors:** Yasuhisa Ano, Makiko Ozawa, Toshiko Kutsukake, Shinya Sugiyama, Kazuyuki Uchida, Aruto Yoshida, Hiroyuki Nakayama

**Affiliations:** 1 Central Laboratories for Key Technologies, Kirin Company Ltd., 1–13–5 Fukuura Kanazawa-ku, Yokohama-shi, Kanagawa, 236–0004, Japan; 2 Graduate School of Agricultural and Life Sciences, the University of Tokyo, 1–1–1 Yayoi, Bunkyo-ku, Tokyo, 113–8657, Japan; 3 Koiwai Dairy Products Co., Ltd., 36–1 Maruyachi, Shizukuishi-cho, Iwate, 020–0507, Japan; Sungkyunkwan University, Republic of Korea

## Abstract

Despite the ever-increasing number of patients with dementia worldwide, fundamental therapeutic approaches to this condition have not been established. Epidemiological studies suggest that intake of fermented dairy products prevents cognitive decline in the elderly. However, the active compounds responsible for the effect remain to be elucidated. The present study aims to elucidate the preventive effects of dairy products on Alzheimer’s disease and to identify the responsible component. Here, in a mouse model of Alzheimer’s disease (5xFAD), intake of a dairy product fermented with *Penicillium candidum* had preventive effects on the disease by reducing the accumulation of amyloid β (Aβ) and hippocampal inflammation (TNF-α and MIP-1α production), and enhancing hippocampal neurotrophic factors (BDNF and GDNF). A search for preventive substances in the fermented dairy product identified oleamide as a novel dual-active component that enhanced microglial Aβ phagocytosis and anti-inflammatory activity towards LPS stimulation *in vitro* and *in vivo*. During the fermentation, oleamide was synthesized from oleic acid, which is an abundant component of general dairy products owing to lipase enzymatic amidation. The present study has demonstrated the preventive effect of dairy products on Alzheimer’s disease, which was previously reported only epidemiologically. Moreover, oleamide has been identified as an active component of dairy products that is considered to reduce Aβ accumulation via enhanced microglial phagocytosis, and to suppress microglial inflammation after Aβ deposition. Because fermented dairy products such as camembert cheese are easy to ingest safely as a daily meal, their consumption might represent a preventive strategy for dementia.

## Introduction

With aged populations growing rapidly around the world, cognitive decline and dementia are becoming an increasing burden not only on patients and their families, but also on national healthcare systems. Alzheimer’s disease is a progressive irreversible brain disorder, symptoms of which include memory loss, confusion, impaired judgment and loss of language skills, and the number of affected individuals is rising sharply. Because cognitive function declines in accordance with an accumulation of Amyloid β (Aβ) in the brain, Aβ deposition is a crucial part of the pathology [1].

Recent pathological and immunological studies revealed that chronic inflammation following Aβ deposition in the brain is closely associated with Alzheimer’s disease pathology [2–4]. Microglia play unique immunological roles including the removal of apoptotic cells and waste such as Aβ through phagocytosis, as well as host defense against virus infection in the central nervous system [5–6]. In the Alzheimer’s brain, however, microglia infiltrate the region around the Aβ plaques, become excessively activated, and produce large amounts of inflammatory cytokines and chemokines, such as tumor necrosis factor-α (TNF-α), macrophage inflammatory protein-1α (MIP-1α), reactive oxygen (ROS) and nitric oxide (NO) [7–9]. These products, which are chronically generated by microglia, after Aβ deposition are toxic to neurons and cause neuronal cell death [7, 10–11].

Numerous reports suggest that controlling microglial activities is effective in the prevention and cure of Alzheimer’s disease and cognitive decline [12–14]. Epidemiological studies suggest that prolonged use of nonsteroidal anti-inflammatory drugs (NSAIDs), including the common medication ibuprofen, significantly reduce the risk for Alzheimer’s disease [15–16]. Consistent with epidemiological research, chronic ibuprofen treatment was found to significantly suppress microglial inflammation and the development of Aβ pathology in a transgenic model mouse of Alzheimer’s disease [17]. However, side effects in the gastrointestinal tract, liver and kidney caused by inhibiting cyclooxygenase I preclude the widespread use of NSAIDs in preventing the disease. Therefore, alternative treatments to Alzheimer’s disease and cognitive decline need to be explored and, as a result, preventive approaches based on diet, exercise and learning are attracting increasing attention.

Several recent epidemiological studies suggest that consumption of fermented dairy products may reduce the risk of cognitive decline in the elderly and prevent dementia including Alzheimer’s disease [18–20]. However, the underlying mechanism and the active compounds remain to be elucidated.

In the present study, the preventive effects of fermented dairy products were evaluated using a transgenic mouse model of Alzheimer’s disease, with a particular focus on microglial activities. In addition, after animal evaluation, compounds in dairy products were screened to identify components capable of enhancing microglial Aβ phagocytosis and anti-inflammatory activity.

## Materials and Methods

### Animals

Pregnant C57BL/6J mice, 8-week-old C57BL/6J male mice, and 6-week-old CD-1 male mice were purchased from Charles River Japan (Tokyo, Japan). Alzheimer’s disease model mice, B6SJL-Tg mice (APPSwFlLon,PSEN1*M146L*L286V, http://jaxmice.jax.org/strain/006554.html, [21]) were purchased from Jackson Laboratory (CA, USA). Mice under 3 months of age were fed with a standard purified rodent growth diet (AIN-93G, Oriental Yeast, Tokyo, Japan), and those over 3 months with a maintenance diet (AIN-93M, Oriental Yeast).

B6SJL-Tg mice were maintained in the experimental facility at the University of Tokyo, and the experiments were approved by the Animal Care and Use Committee of the University of Tokyo and conducted in strict accordance with their guidelines. Pregnant C57BL/6J mice, 8-week-old C57BL/6J mice, and 6-week-old CD-1 mice were maintained in Kirin Company Ltd, and the experiments were approved by the Animal Experiment Committee of Kirin Company Ltd and conducted in strict accordance with their guidelines. All efforts were made to minimize suffering.

### Preparation of *Penicillium candidum*-fermented dairy product sample

*Penicillium (P.) candidum*-fermented dairy products (camembert cheese) were prepared according to a general manufacture procedure. In brief, sterilized milk was fermented with *Lactococcus lactis* to reduce the pH, and treated with calf rennet. The aggregated curds were fermented by *P*. *candidum*. The fermented products were then freeze-dried and delipidated with n-hexane (Wako, Tokyo, Japan) to remove triglyceride. The nutrient composition of the extracted samples was calculated by Japan Food Research Laboratories (Tokyo, Japan).

AIN-93G and AIN-93M diets (Oriental Yeast) containing 2% (w/w) fermented sample were prepared by a conventional procedure, and each diet with or without sample was adjusted to contain the same calories according to the nutrient composition of the extracted sample.

### Analysis of Aβ_1–42_ deposition and chronic inflammation in transgenic murine brain

To evaluate the effects of fermented dairy products on Alzheimer’s disease, 3-month-old transgenic and wild-type female mice were fed a diet with or without fermented sample (2% w/w) for three months (n = 11 mice in each group). At the age of 6 months, the mice were euthanized and the brains were removed. The left hemisphere of the brain was homogenated in RIPA buffer (Wako) with a multi-beads shocker (Yasui Kikai, Osaka, Japan). After centrifugation at 500,000 × g for 20 min, the total protein concentration of the supernatant was measured with a BCA protein assay kit (ThermoScientific, Yokohama, Japan). To quantify Aβ_1–42_ (Wako), MIP-1α (R&D systems, MN, USA), TNF-α (eBiosciences, CA, USA), IL-1β (eBiosciences), synaptophysin (Life Science Inc., FL, USA), BDNF (Promega, WI, USA), GDNF (Promega), and NGF (Promega) in the hippocampus, an appropriate ELISA kit was used. The right hemispheres were fixed in 10% formalin (Wako) and analyzed immunohistochemically using the following specific antibodies: anti-Aβ1–42 (polyclonal, Invitrogen, CA, USA), anti-Iba-1 (polyclonal, Wako), and anti-MIP-α (polyclonal, R&D systems).

### Preparation of lipid extract

The surface and inside of dairy product fermented with or without *P*. *candidum* were crushed in a mortar after lyophilization, and extracted with n-hexane, chloroform and then with methanol. For gas chromatography mass spectrometer (GC/MS) analysis, the lyophilized samples were suspended in methanol containing 1N KOH, and saponified at 80°C for 2 hours. The lipid fraction was obtained as previously described [22] with some modifications. Vehicle (chloroform: water = 10:9) was added to the fraction (final ratio: approximately 1:1:0.9, v/v/v; chloroform:methanol:water), which was then vortexed for 20 min and centrifuged at 3000rpm for 10 min. The lower phase was dried under a stream of N_2_ at 40°C, dissolved in acetone, and filtered through a Polytetrafluoroethylene membrane disc (Millipore, MA, USA).

### GC/MS conditions

The analysis for primary fatty acid amides using GC/MS has been previously described [23]. An Agilent Technologies Network GC/MS system (7890 GC with 5975 mass detector) was used for the analysis with an HP-5MS capillary column (0.25 mm internal diameter, 0.25 μm film thickness, 30 m long, Agilent Technologies). The temperature was initially held at 80°C for 1 min, raised up to 280°C at a rate of 30°C per min, and then held at 280°C for 23 min. The total run time was 30.7 min. Helium was used as a carrier gas with a flow of 1.2 ml/min. Ionization was obtained by electron impact (electron energy, 70 eV). The temperature of the injection port and the transfer line was 250°C. Splitless injection was used with a volume of 1 μl.

### Primary microglia cell culture

Brain cells were obtained from newborn C57BL/6J mice or 6-month-old B6SJL-Tg mice by papaine treatment using a Neural Tissue Dissociation Kit (P) (Miltenyi Biotec, MA, USA). The cells were treated with 2 μg/ml of anti-CD11b antibody conjugated to microbeads (Miltenyi Biotec), and CD11b-positive cells were isolated by magnetic cell sorting (MACS). Isolated cells with more than 90% purity were plated in a poly-D-lysine (PDL)-coated 96-well plate (BD Biosciences, MA, USA) and cultured in DMEM/F-12 (Gibco, CA, USA) medium supplemented with 10% fetal calf serum (Gibco) and 100 U/ml of penicillium/streptomycin (Sigma-Aldrich, MO, USA).

### *In vitro* cytokine production assay

Microglia isolated from newborn C57BL/6J mice and 6-month-old B6SJL-Tg mice were plated at a density of 30,000 per well in a PDL-coated plate, treated with each extracted sample and with fatty acids mainly contained in milk (linoleic acid, linolenic acid, oleic acid, stearic acid, conjugated linoleic acid, Sigma-Aldrich) and oleamide (Sigma-Aldrich) for 12 hours, and then treated with by lipopolysaccharide (LPS, 5 ng/ml, Sigma-Aldrich) and interferon-γ (IFN-γ, 0.5 ng/ml, R&D systems) for 12 hours. After LPS stimulation, supernatants were applied to a TNF-α production assay and cells were evaluated for the expression of cell markers using a FACS Canto II flow cytometer (BD Bioscience) after staining with the following antibodies: 2 μg/ml of anti-CD11b-APC-Cy7 (BD Pharmingen), 1 μg/ml of anti-CD206-PerCP-Cy5.5 (BioLegend, CA, USA), 1 μg/ml of anti-CD68-APC (BioLegend), and 2 μg/ml of anti-CD80-PE (eBiosciences).

To measure intracellular cytokine production, microglial cells were treated with a leukocyte activation cocktail using BD GolgiPlug (BD Biosciences) for 12 hours and with a BD Cytofix/Cytoperm Fixation/Permeabilization kit (BD Biosciences), and then stained with following antibodies: anti-MIP-1α-PE, 2 μg/ml (eBiosciences); anti-TNF-α-FITC, 1 μg/ml (eBiosciences); anti-interleukin-1β (IL-1β)-FITC, 2 μg/ml (eBiosciences); anti-IL-12p40/p70-APC, 2 μg/ml (BD Pharmingen); anti-CD11b-APC-Cy7, 2 μg/ml; and anti-CD206-PerCP-Cy5.5, 1 μg/ml. The cells were analyzed by flow cytometry.

### *In vivo* anti-inflammatory assay

Six-week-old CD-1 male mice were orally given 50 mg/kg of oleamide dissolved in vehicle including 5% ethanol, 5% cremophor and 90% saline, once a day for 3 days. We confirmed that 10 ml/kg oral administration of vehicle did not induce inflammation in the brain (data not shown; n = 7 mice in each group). Thirty minutes after the last administration, the mice were deeply anesthetized with sodium pentobarbital (Kyoritsu Seiyaku, Tokyo, Japan) and injected intracerebroventricularly with 0.25 mg/kg of LPS (L7895, Sigma). LPS or distilled water (for sham-operated controls) was injected by hand into the cerebral ventricle in a volume of 3 μL as described previously [24–27]. Briefly, a micro-syringe with a 27-gauge stainless steel needle, 2 mm in length, was used for micro injection. The needle was inserted unilaterally 1 mm to the right of the midline point equidistant from each eye, at an equal distance between the eyes and the ears and perpendicular to the plane of the skull (anteroposterior, − 0.22 mm from the bregma; lateral, 1 mm from the bregma). LPS was delivered gradually within 30 s. The needle was taken out after waiting 30 s. All mice exhibited normal behavior after they recovered from the anesthetic. Three hours later, the mice were euthanized, the cerebral cortex and hippocampus in one hemisphere were homogenated in RIPA buffer, and the concentration of TNF-α (eBioscience) and MIP-1α (R&D systems) in the supernatants was quantified by ELISA. The hippocampus in the other hemisphere was dissociated with papaine, stained for microglia with the above-mentioned antibodies, and analyzed by flow cytometry as previously described.

### Phagocytosis of 6-carboxyfluorescein-labeled Aβ_1–42_ by microglia

Microglial phagocytosis of 6-carboxyfluorescein-labeled Aβ_1–42_ (Aβ-FAM, AnaSpec, CA, USA) was evaluated by a plate-based assay. Microglial cells isolated from newborn mice were plated at a density of 50,000 cells per well in a PDL-coated 96-well plate and incubated with 500 nM Aβ-FAM for 24 hours after either oleic acid (Sigma-Aldrich) or oleamide (Sigma-Aldrich) pretreatment for 12 hours. After the medium was removed, extracellular Aβ-FAM was quenched with 0.2% trypan blue, pH 4.4. Cellular fluorescence was measured at 485 nm excitation/535 nm emission using a plate reader (Molecular Device, CA, USA).

### *In vivo* phagocytosis assay

Eight-week-old C57BL/6J male mice were orally given 0, 10 or 50mg/kg of oleamide dissolved in vehicle including 5% ethanol (Wako), 5% cremophor (Sigma-Aldrich) and 90% saline (Otsuka) once a day for 3 days (n = 5 mice in wach group). Three hours after the last administration, microglia in the brain were isolated by MACS, and the phagocytotic activity of the microglia was measured using Aβ-FAM as described above. Populations of CD206- and CD11b-positive cells and the expression of cell markers were analyzed using a flow cytometer after staining with 2 μg/ml of anti-CD36-APC antibody (BioLegend), and 2 μg/ml of anti-CD11b-APC-Cy7 antibody (BD Pharmingen).

### Statistical analysis

All values were expressed as the mean ± SEM. Data from the production assays for cytokines, chemokines, neurotrophic factors and synaptophysin, and from *in vitro* antagonist assays were analyzed by two-way ANOVA, followed by the Tukey-Kramer test. Data from the *in vitro* assays were analyzed by one-way ANOVA, followed by Dunnett’s test or Student’s *t* test. All statistical analyses were performed using the Ekuseru-Toukei 2012 software program (Social Survey Research Information, Tokyo, Japan).

## Results

### Intake of the fermented dairy product substantially reduces Aβ burden and inflammation

**Fig 1:**
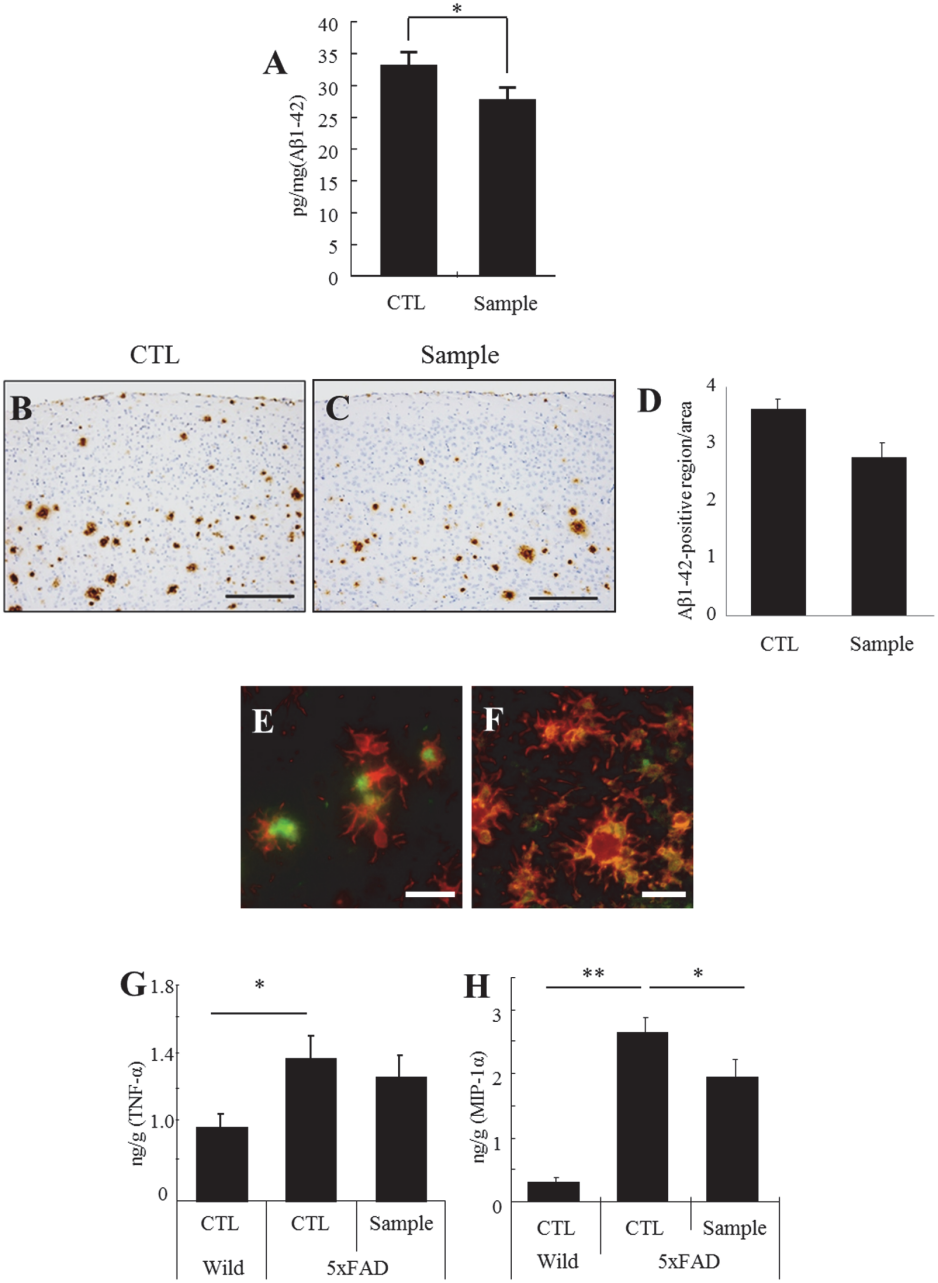
Aβ deposition and inflammation in the brain of mice fed diets with or without dairy product fermented with *P*. *candidum*. Three-month-old 5xFAD transgenic mice were fed a control diet (CTL) or a diet containing 2% (w/v) extract (sample) for 3 months. ***A***, Quantification of soluble Aβ_1–42_ in the brain by ELISA. ***B and C***, Immunohistochemical detection of Aβ_1–42_ distribution in the cerebral cortex. ***D***, Immunohistochemical analysis of Aβ_1–42_ distribution in the cerebral cortex. ***E and F***, Immunofluorescent detection of Iba-1-positive microglia (red), and either Aβ_1–42_ or MIP-1α (green), respectively. ***G and H***, Quantification of cytokines (TNF-α) and chemokine (MIP-1α) in the hippocampus by ELISA. Error bars represent the means ± SEM of 11 mice per group. **p < 0.01 and *p < 0.05. Scale bars represent 200 μm (B and C) and 20 μm (E and F)

The amount of soluble Aβ_1–42_ in the brain of 5xFAD transgenic mice was significantly decreased by 17% in the group fed with the dairy sample (Fig. 1*A*). The insoluble Aβ_1–42_ in cerebral cortex detected immunohistochemistry was decreased by 21% in the sample group but the reduction was not significant (Fig. 1*B*, *C*, *D*). Pathological staining examination by immunohistochemistry revealed that the Aβ1–42 burden was reduced by 21% in the cerebral cortex but 12.5% in the hippocampus. Iba-1-positive microglia had massively infiltrated and engulfed Aβ_1–42_ (Fig. 1*E*), and produced MIP-1α (Fig. 1*F*). The enhanced production of MIP-1α in the transgenic mice was significantly suppressed by administration of the dairy product (Fig. 1*H*).

### Intake of the fermented dairy product increases hippocampal neurotrophic factors and synaptophysin

**Fig 2:**
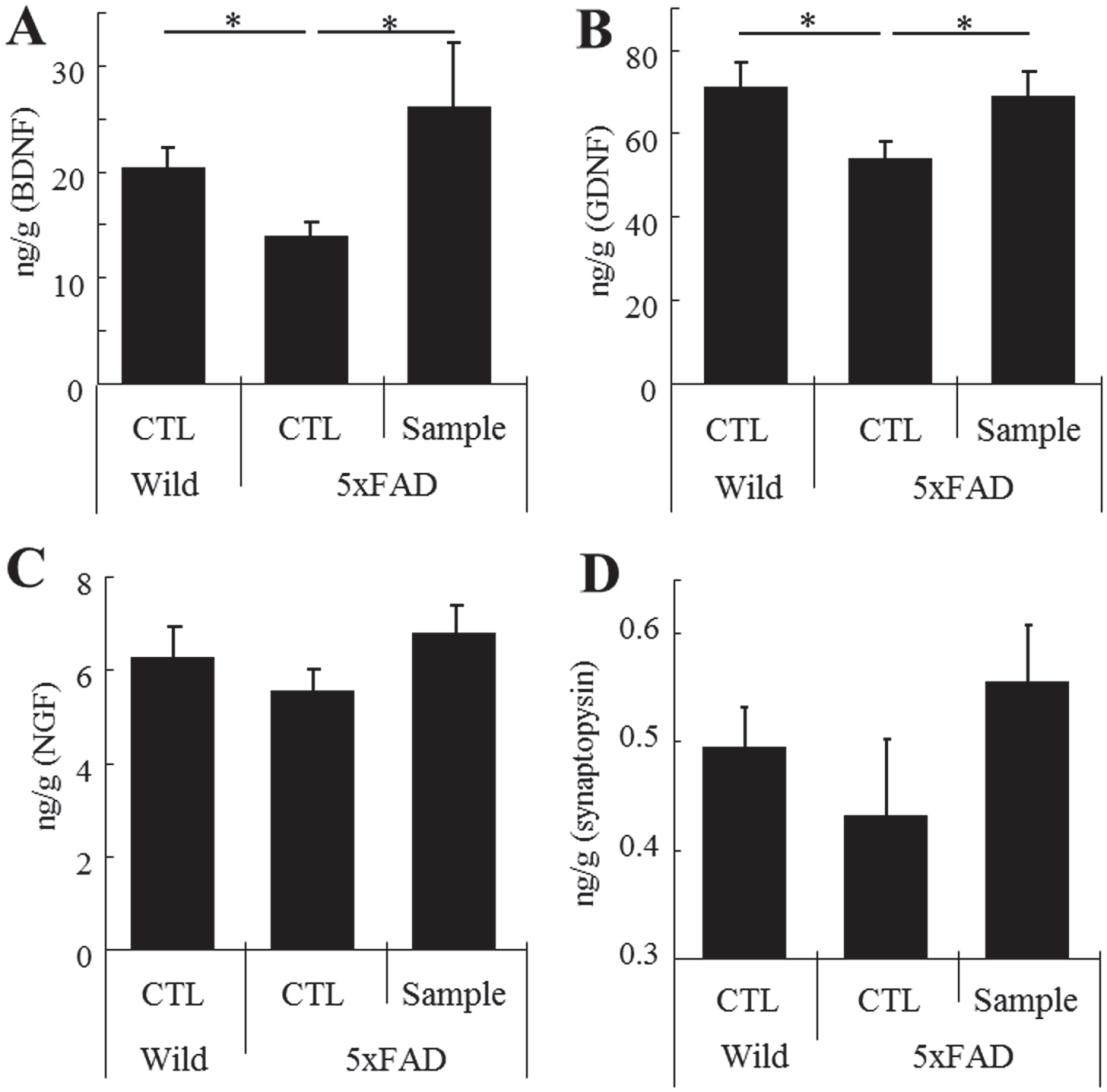
Quantification of neurotrophic factors and synaptic markers in the hippocampus by ELISA. ***A*, *B and C***, Measurement of BDNF, GDNF and NGF, respectively, in the hippocampus. ***D***, Measurement of synaptophysin in the hippocampus. Error bars represent means ± SEM of 11 mice per group. *p < 0.05.

The production of BDNF and GDNF in the hippocampus was significantly lower in the transgenic mice than in wild-type mice. In contrast, the production of these factors was significantly higher in the mice fed with dairy product than in the control mice (Fig. 2*A*, *B*, respectively). The expression of synaptophysin, a neuronal synapse marker was correlated with the productions of neurotrophic factors. (Fig. 2*D*). There were no significant differences in the amount of NGF (Fig. 2*C*) and synaptophysin.

### Anti-inflammatory activity of extracts from the surface of the dairy product

**Fig 3:**
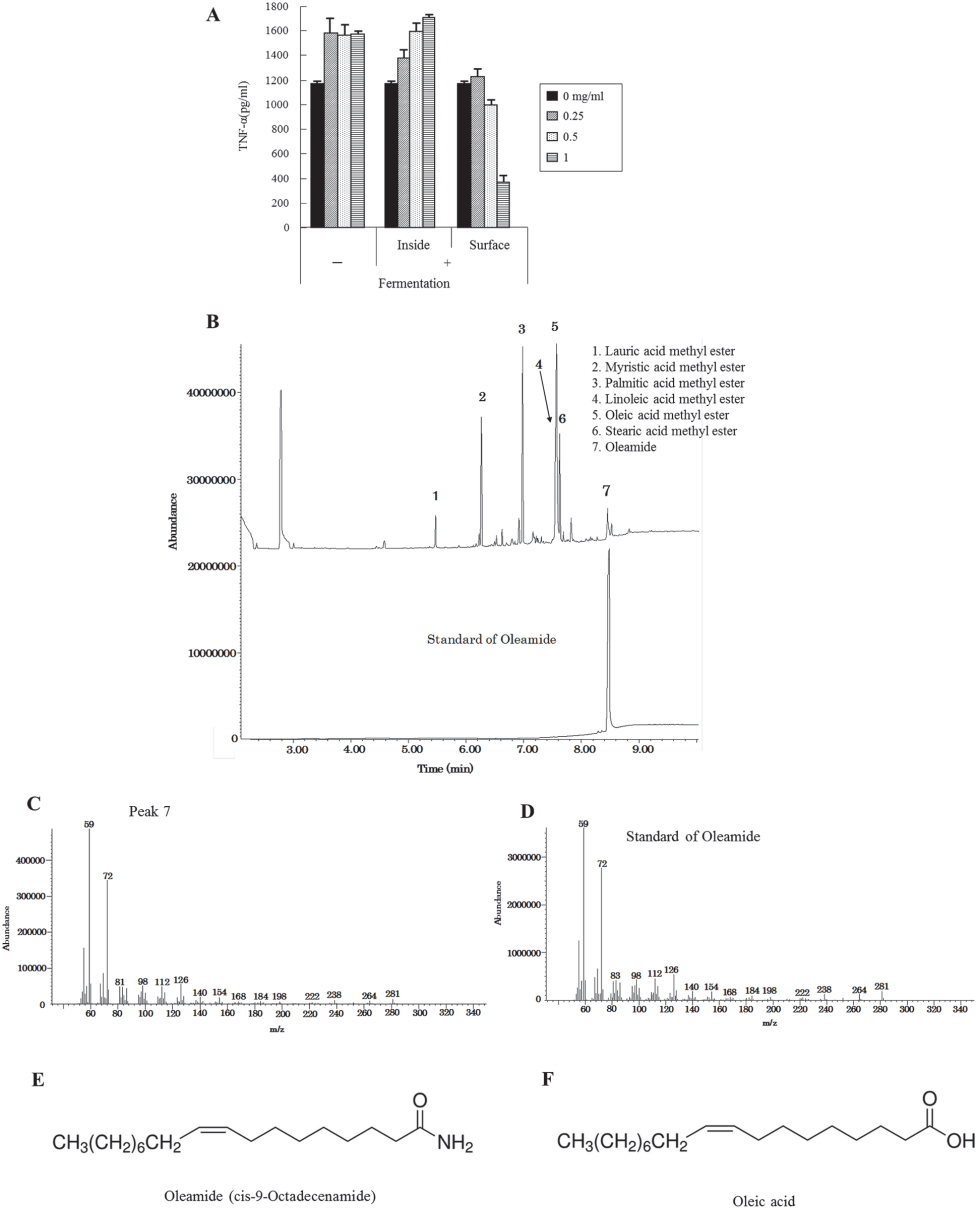
Lipid extraction and GC/MS analysis of extracts from the dairy product fermented with *P. candidum*. ***A***, Isolated microglia were stimulated with 5 ng/ml of LPS and 0.5 ng/ml of IFN-γ after pretreatment with each methanol extract, and TNF-α production in the supernatant was quantified by ELISA. Error bars represent means ± SEM (n = the number of cheese samples) ***B***, Chromatogram of the extract from the surface of the *P*. *candidum*-fermented product and oleamide as a standard. ***C and D***, Mass spectra of Peak 7 and an oleamide standard, respectively. ***E and F***, Chemical structural formula of oleamide and oleic acid, respectively.

The surface and inside part of the dairy product fermented with or without *P*. *candidum* were separately treated with methanol after triglyceride removal by n-hexane and chloroform. The weight of the methanol extract, which mainly contains fatty acids, was increased only on the surface of fermented dairy products (data not shown). The extract from the surface of the fermented dairy product suppressed the production of TNF-α by microglia in a concentration-dependent manner, whereas the extracts from the inside of the product and those without fermentation did not (Fig. 3*A*).

### *P*. *candidum* fermentation generates oleamide

Gas chromatography-mass spectrometry showed a peak identical to oleamide in the extract from the surface of the dairy product fermented with *P*. *candidum* (Fig. 3*B*). The mass spectrum of this peak (Fig. 3*C*) corresponded to the reference standard of oleamide (Fig. 3*D*). Oleamide (Fig. 3*E*) is fatty acid oleic acid (Fig. 3*F*), and was not detected in dairy products that had not been fermented with *P*. *camdidum*.

### Oleamide has microglial anti-inflammatory activity *in vitro*

**Fig 4:**
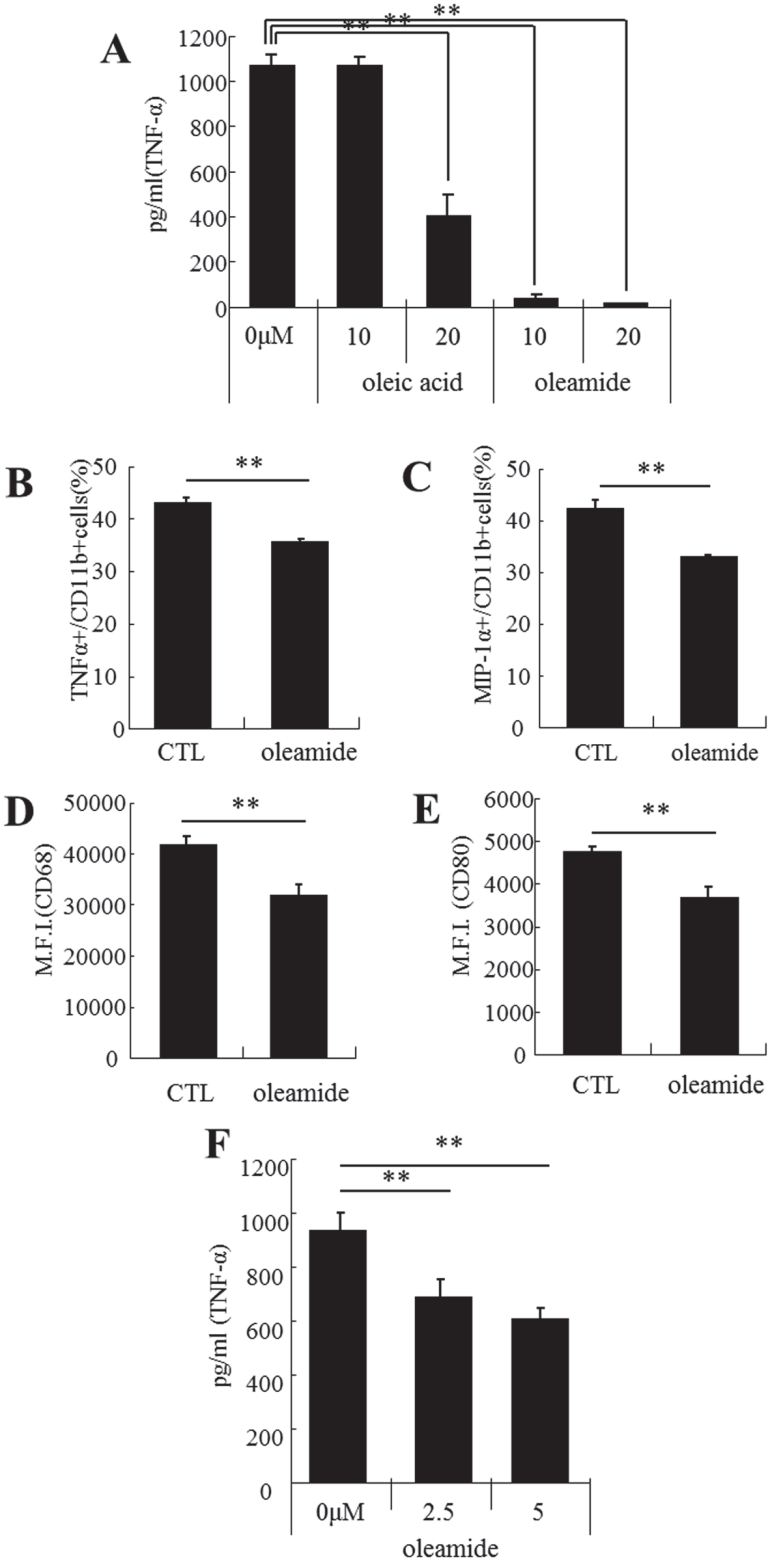
Effect of *in vitro* treatment with oleamide on microglial anti-inflammatory activity. ***A***, Isolated microglia from C57BL/6J newborn were stimulated with 5 ng/ml of LPS and 0.5 ng/ml of IFN-γ after pretreatment with oleic acid or oleamide, and then TNF-α in the supernatant was measured by ELISA. ***B and C***, Isolated microglia were pretreated with oleamide and incubated with PMA, ionomycin, and BD Golgiplug for 12 hours. The ratio (%) of or TNF-α-positive cells or MIP-1α-positive cells to CD11b-positive cells (B and C, respectively). ***D and E***, Expression of CD68 and CD80 in CD11b-positive cells (D and E, respectively). ***F***, Isolated microglia from the brain of 6-month-old 5xFAD transgenic mice were stimulated with 5 ng/ml LPS and 0.5 ng/ml IFN-γ after pretreatment with oleamide, and TNF-α in the supernatant was measured by ELISA. Error bars represent the means ± SEM of 3 wells per group. *p < 0.05 and **p < 0.01.

Oleamide suppressed microglial TNF-α production in a concentration-dependent fashion, and enhanced microglial anti-inflammatory activity more strongly than oleic acid (Fig. 4*A*). The ratios of MIP-1α-positive microglia to CD11b-positive microglia and TNF-α-positive microglia to CD11b-positive microglia were significantly lower after oleamide treatment (Fig. 4*B*, *C*, respectively). Microglia treated with oleamide also showed a lower expression of the inflammatory cell markers CD68 and CD80 in CD11b-positive microglia (Fig. 4*D*, *E*, respectively). In addition, oleamide significantly suppressed TNF-α production in microglia derived from the Aβ-deposited brain of Alzheimer’s disease model mice (Fig. 4*F*).

### Oleamide enhances microglial anti-inflammatory activity *in vivo*

**Fig 5:**
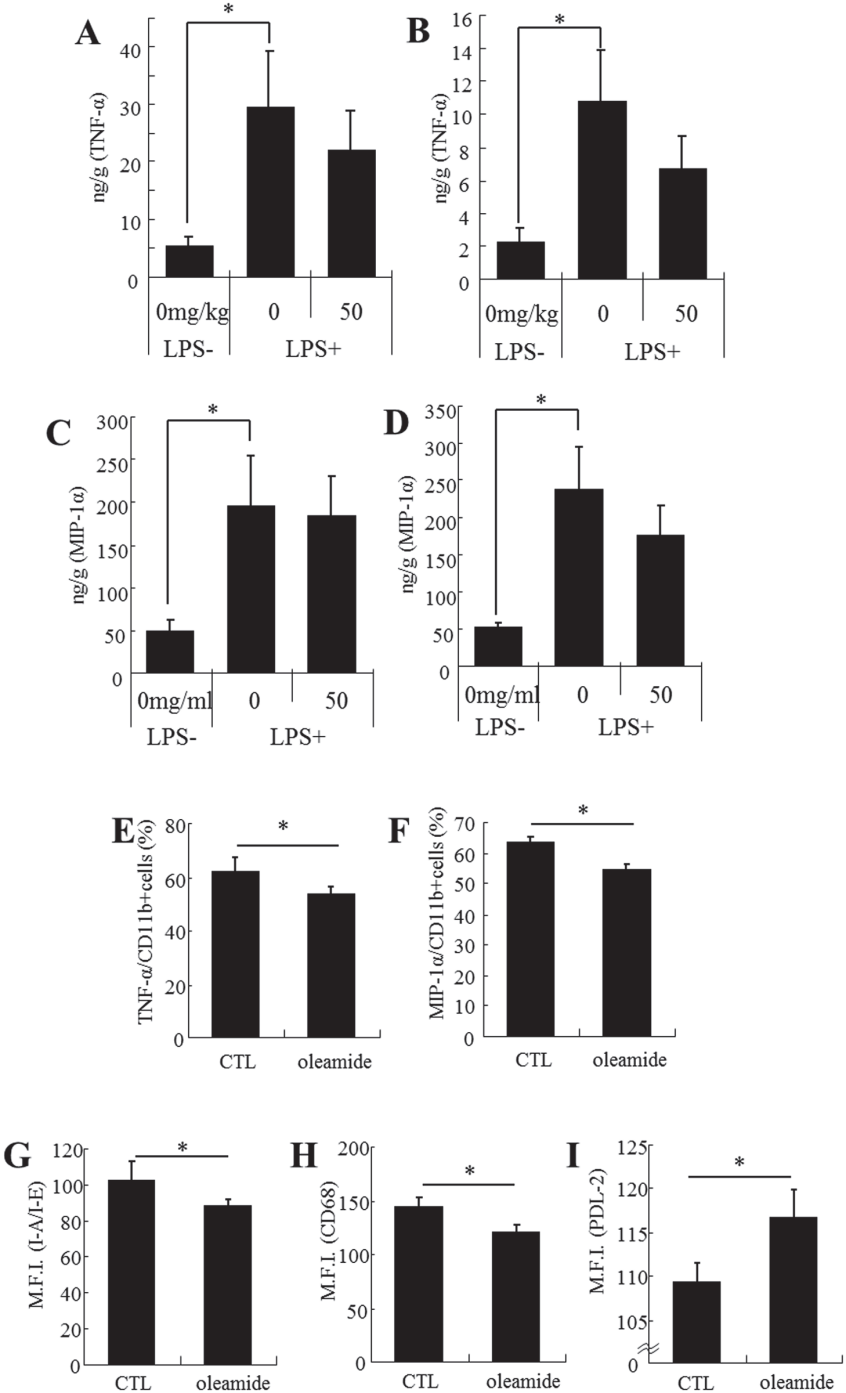
Effect of *in vivo* treatment with oleamide on microglial anti-inflammatory activity. CD-1 mice (n = 7/group) were orally given 50 mg/kg of oleamide once a day for 3 days and then injected intracerebroventricularly with either saline or 0.25 mg/kg of LPS. ***A and B***, Quantification of TNF-α in the cerebral cortex and hippocampus, respectively. ***C and D***, Quantification of MIP-1α in the cerebral cortex and hippocampus, respectively. ***E and F***, The ratio (%) of TNF-α-positive or MIP-1α-positive cells to CD11b-positive cells. ***G*, *H*, *and I***, Expression of I-A/I-E, CD68, and PDL-2, respectively, in CD11b single-positive cells. Error bars represent the means ± SEM of 7 mice per group. *p < 0.05 and **p < 0.01.

Intracerebroventricular injection of LPS significantly increased the production of TNF-α and MIP-1α, but there was no significant difference between the sham and vehicle-treated groups and the group given 50 mg/kg of oleamide and LPS in either the hippocampus (Fig. 5*A*, *C*, respectively) or the cerebral cortex (Fig. 5*B*, *D*, respectively). Flow cytometric analysis revealed that the production of TNF-α and MIP-1α in CD11b-positive microglia was significantly lower in the group administered oleamide (Fig. 5*E*, *F*, respectively). In addition, the expression of I-A/I-E and CD68 was significantly reduced (Fig. 5*G*, *H*, respectively) and that of PDL-2 was significantly increased (Fig. 5*I*) in the group of mice given oleamide.

### Oleamide enhances microglial phagocytosis *in vitro* and *in vivo*

**Fig 6:**
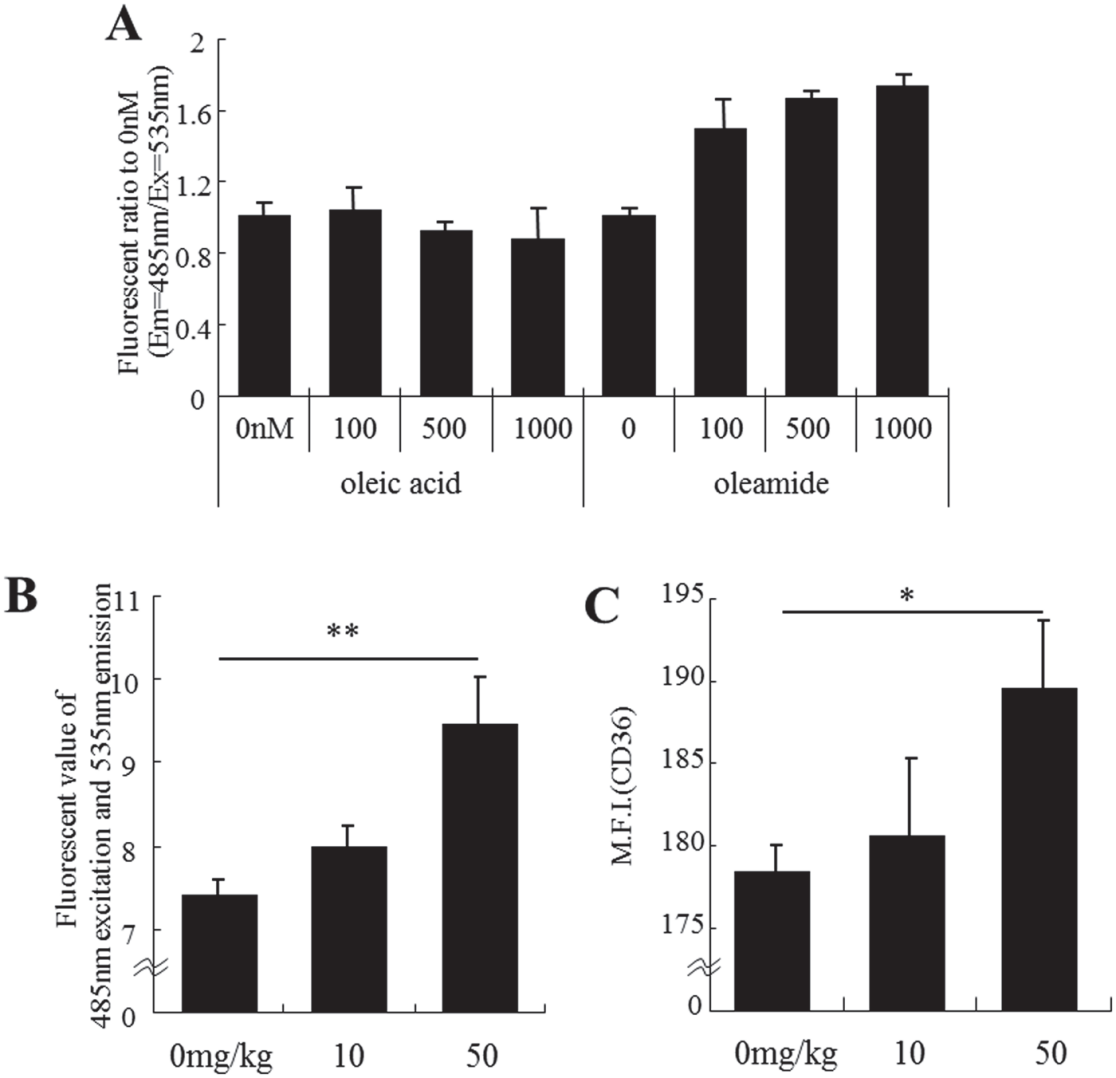
Effect of treatment with oleamide on microglial phagocytosis. ***A***, Aβ phagocytotic activity was measured *in vitro* using isolated microglia pretreated with either oleic acid or oleamide. The value is the ratio of fluorescence to the vehicle. Error bars represent the means ± SEM (n = the number of wells when we examined phagocytosis of Aβ1–42) ***B and C***, Effect of treatment with oleamide *in vivo* on microglial phagocytotic activity. After the oral administration of 0, 10, or 50 mg/kg of oleamide once a day for 3 days and isolation of microglia from the brain, phagocytotic activity and the expression of CD36 were measured. ***B***, Cellular Aβ-FAM was measured after isolated microglia were treated with 500 nM Aβ-FAM for 5 hours and quenched extracellular Aβ-FAM. ***C***, Expression of CD36 in CD11b single-positive cells. Error bars represent the means ± SEM of 5 mice per group. *p < 0.05 and **p < 0.01.

Oleamide at concentrations of 100–1000 nM was found to enhance microglial phagocytosis of Aβ_1–42_ in a concentration-dependent manner *in vitro*, whereas oleic acid did not show activity at all (Fig. 6*A*). In addition, oral administration of 50 mg/kg of oleamide significantly enhanced the brain microglial phagocytosis of Aβ (Fig. 6*B*) and significantly enhanced the expression of CD36 in CD11b-positive microglia in the hippocampus (Fig. 6*C*).

## Discussion

In the present study, the preventive effects of ingesting a fermented dairy product on Alzheimer’s disease, which have previously been reported only epidemiologically, were investigated in a mouse model of Alzheimer’s disease. In mice fed with the fermented dairy product, the deposition of Aβ in the brain was significantly reduced. Microglia play a crucial role in maintaining the brain environment by removing waste products such as amyloid, aged synapses and apoptotic cells via phagocytosis [28–31]. The results of the present study suggest that ingredients in the fermented dairy product contribute to the activation of microglial phagocytosis, resulting in a reduction of Aβ in the brain.

In Alzheimer’s disease, chronic inflammation in the brain exacerbates the pathological condition and cognitive decline because inflammation is toxic to neurons and suppresses the production of neurotrophic factors such as BDNF, GDNF and NGF [32–35]. In the present study, the inflammation induced by Aβ deposition in the transgenic mice was suppressed when the mice were fed with the fermented dairy product. In addition, the production of chemokine (MIP-1α) was remarkably increased in the hippocampus of the transgenic mice and significantly reduced by ingestion of the fermented dairy product. It has been reported that MIP-1α and TNF-α produced by microglia exacerbate the Alzheimer’s pathology [36–37]. The production of both BDNF and GDNF in the hippocampus was also significantly reduced in the model mouse, whereas their production in mice fed with the fermented dairy product was significantly recovered to the levels observed in wild-type mice. It has been suggested that suppression of inflammation contributes to the production of neurotrophic factors, the survival of neuronal synapses and the retention of cognitive function. In the brain, immunological phenomena are mainly regulated by microglia; as a result, it seems likely that some components of the fermented dairy product may regulate the microglial inflammatory response, leading to a suppression of the pathology.

In the transgenic mouse model of Alzheimer’s disease, intake of the fermented dairy product showed preventive effects on development of the disease. Phagocytosis of Aβ by microglia may be essential for clearance of Aβ in Alzheimer’s disease; however, excessively activated microglia also produce several neurotoxic products, such as ROS, NO, cytokines and chemokines, leading to neuronal death [7–9]. Therefore, our results suggest that some ingredients in the fermented dairy product may enhance microglial anti-inflammatory activity and phagocytosis.

Our findings revealed that a certain dairy substance generated during fermentation with *P*. *candidum* is crucial for microglial regulation because the fatty acid extract had high anti-inflammatory effects. In general, linoleic acid and linolenic acid in dairy products are known to have an anti-inflammatory effect. In the present study, linoleic acid and linolenic acid were also identified, but oleamide as a novel ingredient with anti-inflammatory properties was identified from the dairy product fermented with *P*. *candidum*. As compared with linoleic acid and linolenic acid, oleamide has much higher anti-inflammatory activity. In addition, oleamide was identified as a potent dual-active component capable of enhancing both phagocytosis and anti-inflammatory activity. Oleamide is also known as an endogenous substance that binds to cannabinoid (CB) receptors as an agonist [38–39]. However, no previous studies have reported that dairy products contain oleamide. Oleamide is the amide of oleic acid, and is synthesized from oleic acid and ammonia by enzymatic amidation [40]. Oleic acid is abundant in dairy products, and ammonia is generated during the fermentation and maturation of cheese. It is also reported that oleamide is converted from oleic acid via the amidation activity of lipase in a bacteria [41]. These reports suggest that oleamide is generated in the dairy product from oleic acid during fermentation with *P*. *candidum*. In support of this, in the present study oleamide was detected mainly on the surface part of camembert cheese, where *P*. *candidum* grows well, but was not detected in the dairy product without *P*. *candidum* fermentation.

As mentioned above, oleamide has high anti-inflammatory activity. A previous study using an LPS-stimulated murine microglial cell line (BV-2) showed that oleamide suppressed the production of NO and PGE2 [42]. Another study revealed that oleamide suppressed microglial NO production via CB2 receptors, which are mainly expressed on the surface of immune cells (monocytes, macrophages, and B cells). In the present study, oleamide suppressed the productions of MIP-1α and TNF-α, which were predominantly produced in the brain of the Alzheimer’s model mice. Oleamide was also found to have an *in vivo* anti-inflammatory effect in the brain of the Alzheimer’s model mouse. In addition, orally administered oleamide was found to have a potent anti-inflammatory activity on microglia in the LPS-stimulated brain by suppressing the inflammatory cell markers I-A/I-E and CD68, up-regulating the anti-inflammatory cell marker PDL-2, and reducing the inflammatory factors MIP-1α and TNF-α. Therefore, the regulation of microglial activity with oleamide might contribute to the effective prevention of Alzheimer’s disease.

On the other hand, oleamide activates PPAR-γ receptors in cells [43], and its agonist enhances the phagocytotic activity of monocytes [44]. PPAR-γ has recently been receiving increasing attention as a target for Alzheimer’s disease therapy [45–47], so activation of PPAR-γ might be involved in the enhancement of microglial phagocytosis by oleamide. Further studies will elucidate the mechanism of oleamide activate microglial phagocytosis. Oleic acid has no effects on microglia phagocytosis; therefore, amidation of oleic acid must be crucial for this activity. In addition, orally administered oleamide enhanced microglial phagocytosis in the brain and increased the expression of CD36, which is important for uptake of Aβ [46].

In summary, it has been revealed that oleamide in fermented dairy product enhances microglial phagocytosis and suppresses inflammation. Oleamide might prevent Alzheimer’s disease by regulating microglia to a phenotype preferable for maintaining the brain environment. Although camembert alone might not prevent dementia, a well-balanced diet that combines camembert with agreeable foods like red wine or fish is expected to reduce the risk of dementia. Red wine and fish have been proven to contain resveratrol and omega-3 fatty acids respectively, which are beneficial for preventing Alzheimer’s disease. Recent reports suggesting that a Mediterranean diet is associated with lower risk of dementia are supportive of a food culture that combines dairy products with wines and fish dishes [48–49]. Fermented dairy products such as camembert cheese are easy and safe to consume frequently, and might helpful for dementia prevention.
